# Deep‐GB: A novel deep learning model for globular protein prediction using CNN‐BiLSTM architecture and enhanced PSSM with trisection strategy

**DOI:** 10.1049/syb2.12108

**Published:** 2024-11-08

**Authors:** Sonia Zouari, Farman Ali, Atef Masmoudi, Sarah Abu Ghazalah, Wajdi Alghamdi, Faris A. Kateb, Nouf Ibrahim

**Affiliations:** ^1^ National Engineering School of Sfax University of Sfax Sfax Tunisia; ^2^ Department of Computer Science Bahria University Islamabad Campus Islamabad Pakistan; ^3^ Department of Computer Science College of Computer Science King Khalid University Abha Saudi Arabia; ^4^ Department of Informatics and Computer System College of Computer Science King Khalid University Abha Saudi Arabia; ^5^ Department of Information Technology Faculty of Computing and Information Technology King Abdulaziz University Jeddah Saudi Arabia; ^6^ Family Medicine Clinic Makkah Armed Force Medical Center Makkah Saudi Arabia

**Keywords:** bioinformatics, biological techniques

## Abstract

Globular proteins (GPs) play vital roles in a wide range of biological processes, encompassing enzymatic catalysis and immune responses. Enzymes, among these globular proteins, facilitate biochemical reactions, while others, such as haemoglobin, contribute to essential physiological functions such as oxygen transport. Given the importance of these considerations, accurately identifying Globular proteins is essential. To address the need for precise GP identification, this research introduces an innovative approach that employs a hybrid‐based deep learning model called Deep‐GP. We generated two datasets based on primary sequences and developed a novel feature descriptor called, Consensus Sequence‐based Trisection‐Position Specific Scoring Matrix (CST‐PSSM). The model training phase involved the application of deep learning techniques, including the bidirectional long short‐term memory network (BiLSTM), gated recurrent unit (GRU), and convolutional neural network (CNN). The BiLSTM and CNN were hybridised for ensemble learning. The CST‐PSSM‐based ensemble model achieved the most accurate predictive outcomes, outperforming other competitive predictors across both training and testing datasets. This demonstrates the potential of harnessing deep learning for precise GB prediction as a robust tool to expedite research, streamline drug discovery, and unveil novel therapeutic targets.

## Graphical abstract

(1) Introduces Deep‐GP model for accurate identification of globular proteins. (2) Employs a novel feature descriptor called CST‐PSSM. (3) Utilises an ensemble of BiLSTM and CNN for model training.

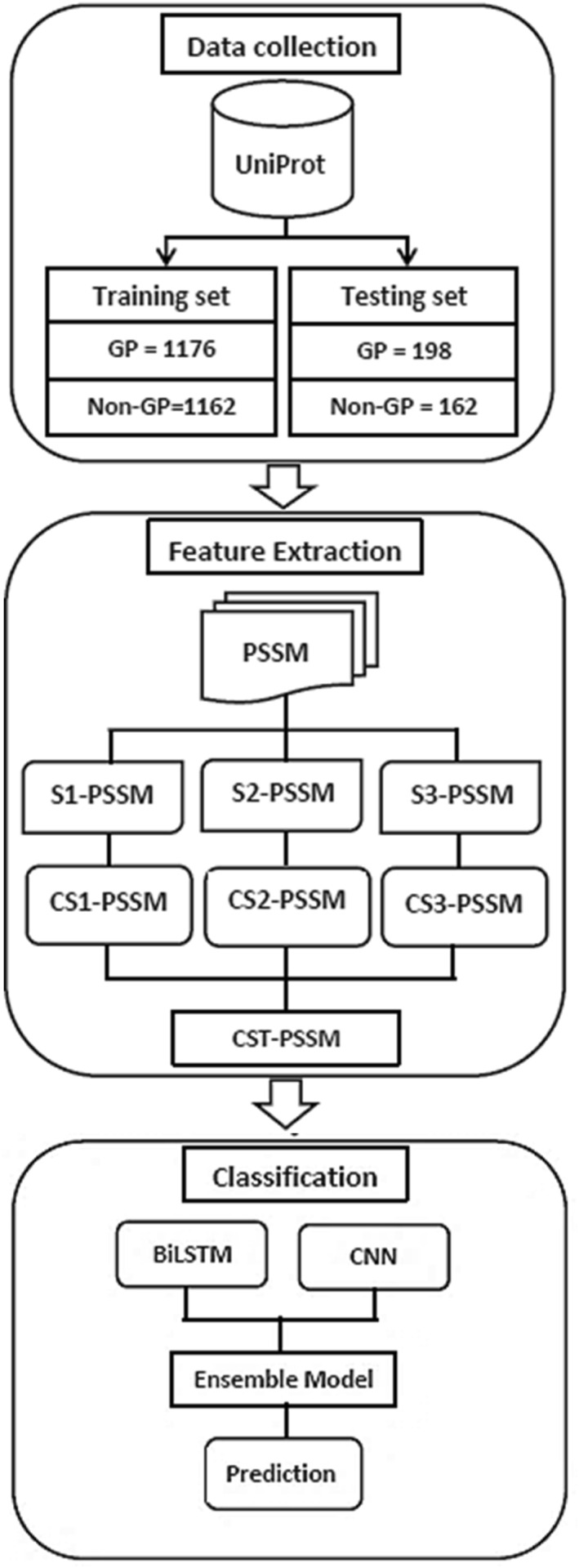

## 1 INTRODUCTION

Globular proteins constitute a category of proteins that play vital roles across various domains, including bodily functions [1], disease treatment [2], and drug discovery [3]. These proteins exhibit a distinctive three‐dimensional structure that facilitates their performance of specific functions within living organisms.

Globular proteins perform active role in a wide array of cellular functions and biological processes. For instance, certain globular proteins act as catalysts for biochemical reactions within the body. Enzymes, a type of globular protein, enable the breakdown of food, the synthesis of crucial molecules, and the regulation of metabolic pathways [4]. Haemoglobin, another example of a globular protein, transports oxygen from the lungs to body tissues, playing a pivotal role in respiratory gas transportation. Furthermore, globular proteins contribute to immune responses by functioning as antibodies and recognising and neutralising foreign substances, such as pathogens or toxins [5].

In drug discovery, globular proteins represent valuable targets for the development of therapeutic approaches. It is essential to comprehend the function of these target proteins for the design of drugs that can selectively interact with them and regulate their activity. The study of the three‐dimensional structure of globular proteins enables researchers to pinpoint potential binding sites for drug molecules [6]. This understanding serves as the foundation for crafting small molecules or biologics capable of interacting with specific globular proteins, either by inhibiting their activity or enhancing their function. The targeting of globular proteins in drug discovery has resulted in the production of numerous life‐saving medications spanning various therapeutic areas, such as neurological disorders [7], cardiovascular diseases [8], and cancer [9].

In cancer, globular proteins can act as oncogenes, promoting uncontrolled cell growth and division. These oncogenes can be mutated or overexpressed, leading to dysregulation of cell cycle control and abnormal cell proliferation. Examples of oncogenic globular proteins include p53, RAS, and MYC. Globular proteins can also act as tumour suppressors, inhibiting cell growth and preventing cancer development. These tumour suppressors can be mutated or lost, leading to uncontrolled cell proliferation and tumour formation. Examples of tumour suppressor globular proteins include Rb, p21, and PTEN [10].

Considering the above significance, identifying GP accurately is crucial in scientific research. However, according to our best knowledge, no computational predictor has developed for identification of GP. This research presents Deep‐GP, a pioneering computational model designed to precisely identify GP.

In this work, we first constructed new sequence‐based training and testing datasets. Second, features from these datasets were extracted by developing a descriptor (CST‐PSSM). Third, model training was performed by integrating the BiLSTM and CNN. Fourth, the model was validated using the five‐fold cross‐validation method. The major contributions of the proposed work are listed below.

- Development of a novel computational model for accurate GP identification.
- Creation of two original primary sequence‐based training and testing datasets.
- Introduction of a new feature descriptor, CST‐PSSM, to effectively capture relevant sequence information.

**FIGURE 1:**
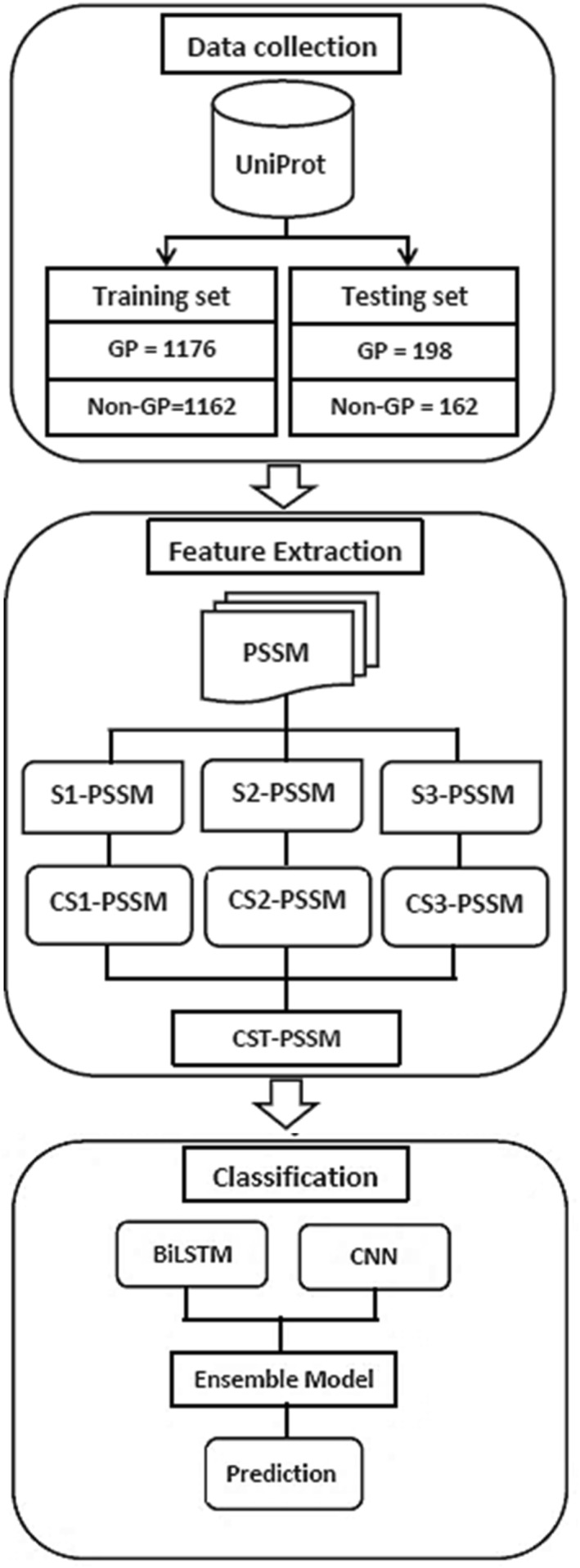
Flow diagram of the proposed study. In the first phase, the collected dataset from the Uniprot database is split into training and testing subsets. In the second step, each PSSM is divided into three sections, consensus sequences are computed from each section, and finally, they are fused to create one superset. In the third step, ensemble learning is carried out using BiLSTM and CNN to perform prediction.

The schematic view of the applied approaches is depicted in Figure 1.

## 2 MATERIAL AND METHODS

### 2.1 Datasets collection

To ensure the robustness of our protocol, we meticulously curated a reliable dataset. We generated this dataset utilising sequences from both globular proteins (GP) and non‐globular proteins (GP). Our dataset comprises two classes: the positive class, encompassing sequences of globular proteins (GP), and the negative class, including sequences of non‐globular proteins (GP).

To compile the samples for our dataset, we conducted a keyword search for “globular protein” on the UniProt database (https://www.uniprot.org), yielding a total of 1435 globular protein sequences and 1429 non‐globular protein sequences. To enhance the dataset's quality, we utilised the CD‐HIT tool [11], which eliminates redundancy by applying a 25% sequence similarity index. Additionally, sequences containing fewer than 50 amino acids were excluded. After these filtration steps, our final dataset comprised 1374 GP sequences and 1344 non‐GP sequences.

To assess our protocol's performance, we randomly divided the dataset into training (80%) and testing (20%) subsets. The training set consisted of 1176 GP and 1162 non‐GP, while the testing set comprised 198 GP and 162 non‐GP. Using the training set, we trained our computational model to recognise the patterns and features present in the dataset.

Subsequently, we utilised the testing set to evaluate the model's ability to generalise. This evaluation enables us to determine the effectiveness of our protocol in distinguishing between GP and non‐GP sequences.

### 2.2 Feature generation methods

To facilitate the application of deep or machine learning frameworks, feature extraction methods are used to encode primary sequences into numerical representation [12, 13, 14]. In this work, we designed a novel descriptor called CST‐PSSM.

#### 2.2.1 Position specific scoring matrix

Composition features (amino acid frequency) can overlook crucial information in protein sequences, particularly global features (long‐range dependencies) and local sequence patterns (short‐range dependencies). Feature extraction techniques such as in studies [15, 16, 17] capture global features by identifying conserved amino acids across similar protein sequences. This aligns with the concept of PSSM that capture evolutionary information. PSSM is a fundamental concept in the field of protein sequence analysis. PSSM is a mathematical representation of the evolutionary conservation of amino acids at each position in a sequence alignment [18, 19]. It serves as a valuable tool for identifying conserved regions, understanding protein structure‐function relationships, and predicting functional elements within a protein or nucleotide sequence [20, 21].

The construction of a PSSM involves analysing a set of homologous sequences, which are sequences derived from different organisms that share a common ancestry [22, 23]. These sequences are aligned to highlight the similarities and differences at each position [24, 25]. The resulting alignment is then used to generate a scoring matrix that reflects the likelihood of finding a particular amino acid at each position based on the observed frequencies in the homologous sequences [26, 27].

The key components of a PSSM include position‐specific substitution scores, which quantify the probability of observing a specific amino acid at a given position [28, 29]. Positive scores indicate a preference for a particular amino acid, suggesting evolutionary conservation, while negative scores indicate less conserved positions [30, 31]. The PSSM is a 20×*L* matrix, where *L* and 20 represent the length and number of amino acids, respectively, in the protein sequence. The following formula is used to formulate the PSSM.

(1) $$PSSM={\left(P_{1},P_{2},\text{…}..,P_{j},\text{…}..,P_{20}\right)}^{T}$$

(2) $$P_{i,j}=\left(P_{1,j},P_{2,j},\text{…}..,P_{L,j}\right),\left(\;i=1,2,\text{…}..,L\right)$$

where the transpose operator is denoted by T. The score given to the amino acid at the *i*th position of the query sequence that mutated to the *j*th amino acid type during the evolutionary process is indicated by the element $P_{i,j}$.

#### 2.2.2 Consensus sequence‐based trisection PSSM

In the encoding process of protein sequences, global features, which provide crucial information, are often ignored using the composition feature group [32, 33, 34]. To preserve information about protein sequence length, the consensus sequence (CS) concept is integrated into PSSM. This integration involves deriving consensus sequences from evolutionary data by substituting amino acids in the original sequence with those exhibiting the highest substitution probability [35, 36]. This two‐step process begins by identifying the amino acid with the maximum substitution probability at each position within the sequence.

(3) $$P_{m}=\mathrm{argmax}\left(S_{m,n}:1\leq n\leq 20\right),1\leq m\leq L$$

The symbol $S_{m,n}$ denotes the substitution probability of the amino acid at position *m* with the *n*th amino acid in the PSSM. Subsequently, replace the amino acid at the *m*th position of the original protein sequence with the $P_{m}^{th}$ amino acid to create the consensus sequence. Based on the information in the PSSM, compute the frequency of each amino acid inside the sequence and construct a 20‐feature vector from the consensus sequences.

The Trisection PSSM (TS‐PSSM) is a feature encoding strategy rooted in PSSM, aiming to systematically delve into evolutionary information [36, 37]. Although PSSM captures evolutionary information, the traditional feature encoding process from PSSM can disregard correlations between amino acid residues and local sequence patterns [38]. To preserve this information, we propose the Trisection PSSM method. This approach divides the PSSM into three equal‐sized sections. Each resulting S‐PSSM can be described as follows:

(4) $$S-PSSM\left(\xi \right)={\left[\begin{array}{cccc} P_{r+1,1} & P_{r+1,2} & \text{…}. & P_{\begin{array}{c} r+1,20 \end{array}}\\ P_{r+2,1} & P_{r+2,2} & \text{…}. & P_{\begin{array}{c} r+2,20 \end{array}}\\ \text{⋮} & \text{⋮} & \text{⋮} & \text{⋮}\\ P_{r+N\left(\xi \right),1} & P_{r+N\left(\xi \right),2\;} & \text{…}. & P_{r+N\left(\xi \right),20} \end{array}\right]}_{N\left(\xi \right)\times 20}$$

Here *r* = (*ξ* − 1)*N(*ξ*), *ξ* = 3 is the number of S‐PSSM, and *N*(*ξ*) denotes rows in each S‐PSSM. Similarly, ⌊∗⌋ operator is the rounding down.

Both global and local features possessed effective information within a protein sequence [32]. Recognising this, we employed CS‐PSSM to capture the global motifs and TS‐PSSM to focus on local regional patterns [38, 39, 40]. However, we wanted to leverage the best of both approaches. So, we developed CST‐PSSM, a novel feature representation approach that combines the strengths of CS‐PSSM and TS‐PSSM. This innovative method allows us to extract highly discriminative information from protein sequences, boosting our ability to analyse them effectively. In this method, each PSSM is split into three sections. Consensus sequences are derived from each section of PSSM using Eq. (3). Finally, we combine features of all the sections which is expressed as follows:

(5) $$CST-PSSM={\left[S-PSSM\left(1\right)+S-PSSM\left(2\right)+S-PSSM\left(3\right)\right]}_{1\times \left(20\right)}$$

With this method, we generated a feature vector of 60 dimension.

### 2.3 Deep learning frameworks for model training

Following feature extraction, a powerful algorithm is vital for optimal performance. We compared various deep learning and machine learning techniques (BiLSTM, GRU, CNN, integrated CNN + BiLSTM, Extremely Randomised Tree (ERT), Random Forest (RF), and Extreme Gradient Boosting (XGB)). The CNN + BiLSTM significantly boosted the study's overall performance, and its details are provided in the following sections.

#### 2.3.1 Hybrid model

Hybrid methods, known for their ability to mitigate individual model weaknesses, were a natural choice for our study. We hybridised two deep learning algorithms that is, BiLSTM and CNN. This synergistic approach harnesses the complementary strengths of each model, reducing bias and variance instability.

##### Convolutional neural network

CNN architectures, widely adopted for classification tasks [41, 42] typically comprise input, hidden, and output layers. The hidden layer is composed of sub‐layers including zero‐padding, convolution, pooling, and fully connected layers, which process the input feature vectors [43]. To address potential information loss when using filters during convolution, a zero‐padding layer is applied to all sides of the input data, preserving the original data dimension [44]. Subsequently, a convolutional layer is applied, that performs the convolution operation and an activation function. The convolution operation, a key computational component, slides over the input data to construct feature maps at each position [45]. CNN incorporates multiple convolutional layers, each executing convolution operations using distinct filters.

The output proceeds through a max‐pooling layer with a 2×2 stride. Max‐pooling reduces spatial dimensions, lowers parameters, controls overfitting, and reduces training time [46]. Fully connected layers, the final hidden layers, learn non‐linear feature patterns for classification. Dropout layer is incorporated after fully connected layers to mitigate overfitting and enhance classification accuracy. Dropout deactivates a random fraction of neurons during training, enabling the model to make predictions based on diverse features.

**FIGURE 2:**
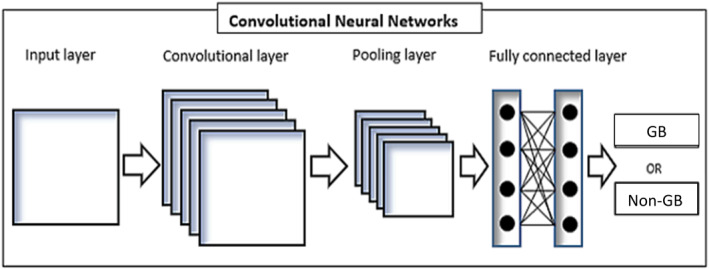
Typical view of the CNN structure.

The output layer includes a flatten layer, converting features into vector shapes, and a sigmoid layer that outputs probabilities for each outcome. The simple CNN structure is shown in Figure 2.

In our study, three convolution layers were utilised to optimise performance with varying filter counts (16–256), with the model achieving optimal results using 64 filters. CNN architectures employ various optimisers such as Adagrad, Adadelta, RMSprop, and Adam, with Adam yielding the best results. Additional hyperparameters included in our model are detailed in Table 1.

**TABLE 1:** List of hyperparameters of the CNN model.

Hyperparameter	Value
Optimiser	Adam
Activation function	Sigmoid
Convolution layers	3
Learning rate	0.001
Dropout	0.4
Batch size	70
Number of epochs	30

##### Bidirectional long short‐term memory network

BiLSTM is a powerful tool for analysing sequential data. Building on the traditional Long Short‐Term Memory (LSTM) network, BiLSTM revolutionises the field by processing data in both forward and backward directions. This unique approach unlocks hidden patterns and dependencies within sequences [47].

LSTM is known for its ability to handle sequential data, making it ideal for time series [48], natural language processing [49], and bioinformatics [50]. However, they struggle to grasp the full context surrounding a specific data point, as they only consider the past. BiLSTM tackles this limitation by processing data in both directions, allowing it to not only learn from past information but also anticipate future input. This “double‐vision” approach creates a richer representation of dependencies, enabling BiLSTM to capture long‐range contextual information that's crucial for tasks, such as protein sequence analysis [51].

BiLSTM considers both past and future contexts proves invaluable. It helps understand the intricate relationships between amino acids within a protein sequence, leading to more accurate predictions. This enhanced understanding arises from the improved information flow during feature learning, ultimately boosting the network's discrimination power [52].

**FIGURE 3:**
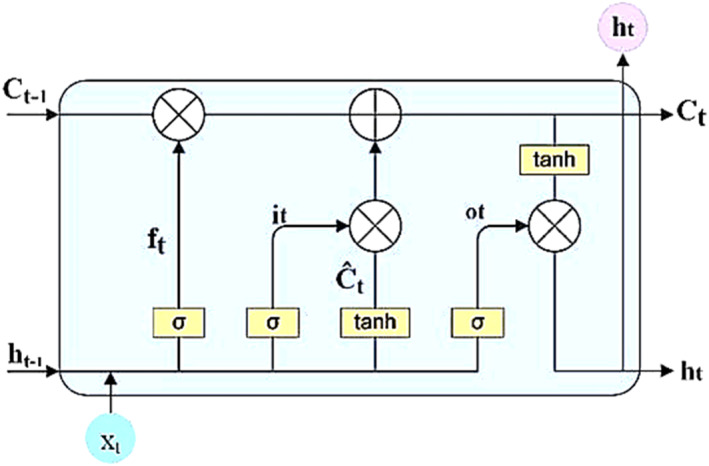
Structure of single LSTM unit.

While the inner workings of a single LSTM unit are detailed in Figure 3, the key takeaway is that BiLSTM's bidirectional processing unlocks a deeper level of understanding of sequential data, making it a go‐to tool for diverse applications. This retains the key points of the original data but simplifies the language, removes repetition, and focuses on the practical advantages of BiLSTM.

When input data is received, the LSTM unit undergoes a three‐step process. Initially, the determination of information to retain is made by the “forgetting gate” and the output from the previous time step. In the second step, two processes occur the generation of new information through the “input gate” and the addition of this new information to update the current unit state via a tanh layer. Finally, in the third step, the LSTM unit generates its output using the “output gate” and a tanh layer [53].

### 2.4 Model assessment approaches

A newly developed predictor is validated using a robust method to assess its performance and achieve the desired output [54, 55]. In this investigation, we employ the widely adopted 5‐fold Cross‐Validation (CV) approach, a methodology mostly used in various biological problems [56, 57, 58, 59, 60]. Within the 5‐fold CV, the dataset is divided into five equal sets. The model is trained on four of these folds and subsequently tested on the fifth, a process repeated five times, with each iteration involving testing on a different fold. The overall performance is determined as the average of all 5‐fold results [61].

Furthermore, performance evaluation metrics such as MCC, Sn, Sp, and Acc are employed. These metrics are derived from the confusion matrix, which tallies correctly and incorrectly predicted instances, and are defined by the formulas below:

(6) Accuracy=(TP+TN)(TP+FP+TN+FN)

(7) Sensitivity=TP(TP+FN)

(8) Specificity=TN(TN+FP)

(9) MCC=(TN×TP)−(FN×FP)(TP+FN)(TP+FP)(TN+FN)(TN+FP)

These assessment metrics are derived from the Confusion Matrix (CM), which comprises four elements: true negative (TN), false positive (FP), true positive (TP), and false negative (FN). These elements are employed to calculate the aforementioned evaluation parameters. TP signifies the accurate predictions of the GP, while TN corresponds to instances of non‐GP correctly identified. FP denotes instances of non‐GP wrongly predicted as GP by the model, and FN represents GP instances incorrectly classified as non‐GP.

## 3 RESULTS AND DISCUSSION

In this section, the performance of several classifiers is assessed utilising different feature descriptors. The subsequent subsections offer a comprehensive comparison of their efficacy.

### 3.1 Performance of deep learning models

The results obtained from various deep learning models utilising GAAC, PSSM, and CST‐PSSM feature representation strategies are presented in Table 2. With the GAAC feature descriptor, the GRU model achieves an accuracy of 79.78%, sensitivity of 80.34%, and specificity of 76.90%. The MCC of 0.61 indicates a moderate overall performance, effectively balancing predictions for both GP and non‐GP occurrences. The BiLSTM model, employing GAAC, outperforms the GRU model in terms of accuracy, sensitivity, MCC, and specificity. The CNN model performs well, securing Acc of 80.31%, Sn of 85.11%, and Sp of 76.32%, and MCC of 0.62, demonstrating a strong ability to distinguish between the two classes. Notably, the CNN + BiLSTM ensemble model surpasses CNN, GRU, and BiLSTM on GAAC, with the highest Acc (81.89%), Sn (88.96%), Sp (76.78%), and MCC (0.63).

**TABLE 2:** Performance of the deep learning approaches.

Classifier	Feature descriptor	Acc (%)	Sn (%)	Sp (%)	MCC
GRU	GAAC	79.78	80.34	74.90	0.61
BiLSTM		81.02	87.89	75.85	0.62
CNN		80.31	85.11	76.32	0.60
CNN + BiLSTM		81.89	88.96	76.78	0.63
GRU	PSSM	80.37	83.77	76.98	0.58
BiLSTM		79.88	83.43	75.23	0.57
CNN		81.22	81.66	81.12	0.62
CNN + BiLSTM		83.55	91.17	76.21	0.67
GRU	CST‐PSSM	86.32	87.75	87.91	0.76
BiLSTM		89.34	90.16	89.41	0.79
CNN		90.44	90.99	90.24	0.80
CNN + BiLSTM		91.78	90.83	91.01	0.81

Examining the PSSM approach, GRU on PSSM exhibits better performance than the GAAC‐based model. The GRU and CNN further enhance performance across all evaluation parameters, indicating that PSSM captures meaningful features by utilising evolutionary patterns. CNN + BiLSTM on the PSSM 83.55% Acc, 91.17% Sn, 76.21% Sp, and 0.67 MCC. CNN + BiLSTM consistently shows good results, attributed to BiLSTM's effectiveness in modelling temporal relationships in sequential data and CNN's proficiency in extracting spatial information. Combining them allows the model to leverage both temporal and spatial patterns, providing a deeper understanding of the data.

Considering the CST‐PSSM feature representation approach, GRU's performance is remarkable, yielding an accuracy of 86.32%, which is 6.54% and 5.95% higher than GAAC and PSSM, respectively. BiLSTM also demonstrates better performance compared to GAAC and PSSM, with higher accuracy, sensitivity, specificity, and MCC. Similarly, CNN + BiLSTM with CST‐PSSM improved the Acc, Sn, Sp, and MCC by 89.45%, 91.78%, 90.83%, and 0.81, respectively. Among all classification algorithms, the ensemble model consistently delivers superior performance by capturing hierarchical temporal dependencies, while CNN excels at capturing hierarchical spatial features. Hybridising these models allows to capture hierarchical patterns in both temporal and spatial dimensions, providing meaningful features. In conclusion, the CST‐PSSM feature representation approach proves effective across different deep learning frameworks, demonstrating its ability to extract local features using a slicing strategy while considering global information, making it a unique approach.

### 3.2 Performance comparison with machine learning models

To systematically investigate the performance of various machine learning algorithms, we conducted a series of experiments using consistent feature engineering, validation, and evaluation protocols. The resulting performance metrics are presented in Table 3. Hyperparameter optimisation was carried out using grid search to identify optimal configurations for each algorithm. Analysing the results in Table 3 reveals that RF produced 89.65% Sn and 85.18% Sp. The accuracy and MCC values for RF are 87.28% and 0.73, respectively. ERT demonstrated superior performance with higher accuracy (88.53%), sensitivity (79.98%), specificity (83.27%), and MCC (0.714). On the other hand, XGB outperformed RF and ERT classifiers, generating an accuracy of 88.53%, sensitivity of 89.98%, specificity of 86.92%, and MCC of 0.74. These results reflect the superiority of XGB.

**TABLE 3:** Performance of the machine learning models.

Classifier	Acc (%)	Sn (%)	Sp (%)	MCC
RF	87.28	89.65	85.18	0.73
ERT	88.53	89.98	86.92	0.74
XGB	89.61	87.90	90.87	0.76
Deep‐GB	91.78	90.83	91.01	0.81

**FIGURE 4:**
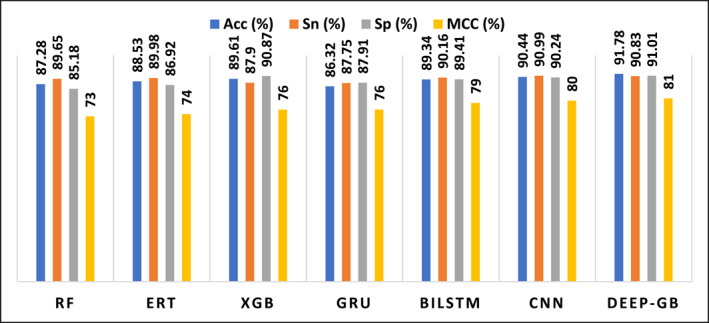
The comparative performance of the proposed study with other applied models.

Our proposed model, Deep‐GB, generated remarkable performance, boosting accuracy to 91.78%. The sensitivity reached 90.83%, indicating the model's efficacy in identifying positive instances (GB). The high specificity of 91.01% contributes to a well‐balanced performance. Deep‐GB demonstrates an excellent overall performance with MCC value of 0.81, signifying its effectiveness in establishing a balance between GB and non‐GB. This high MCC value suggests that Deep‐GB is adept at correctly classifying both positive and negative cases, making it a valuable tool for GB prediction. These results validate the efficacy of our study, highlighting its potential as a robust deep‐learning model for accurately identifying GB. The performance schematic view of the proposed work and other applied models is depicted in Figure 4.

### 3.3 Results comparison on the testing dataset

Subsequent to training phase completion, the model's predictive capabilities were examined using an independent testing dataset to evaluate its generalisation performance. GRU, BiLSTM, and CNN were also utilised in the testing dataset, and the results are summarised in Table 4. The GRU model achieved Acc of 83.94%, Sn of 75.78%, Sp of 92.33%, and MCC of 0.68. The BiLSTM model yielded higher accuracy at 84.34%. When comparing the CNN model to the GRU and BiLSTM models, it improved the values for accuracy, sensitivity, specificity, and MCC.

**TABLE 4:** Performance of models on the testing set.

Classifier	Acc (%)	Sn (%)	Sp (%)	MCC
GRU	83.94	75.78	92.33	0.68
BiLSTM	84.32	76.86	94.11	0.69
CNN	85.27	77.94	94.83	0.70
Deep‐GB	87.63	78.81	95.25	0.72

On the testing dataset, our proposed predictor reflected an accuracy of 87.63%, sensitivity of 78.81%, specificity of 95.25%, and MCC of 0.72. These results validate the precise GB prediction capabilities of Deep‐GB. The findings validate the remarkable performance and promising generalisation efficacy of our predictor, surpassing BiLSTM, CNN, and GRU models on the testing dataset across all assessment parameters. The comparative results of these classifiers are detailed in Table 4, highlighting the superior accuracy of Deep‐GB in predicting GB with precision.

### 3.4 Pros and cons of the proposed study

The proposed computational method exhibits several strengths. Notably, it introduces a novel Deep‐GP model for accurate GP prediction, surpassing existing approaches in performance. The integration of the CST‐PSSM feature descriptor enhances feature extraction, contributing to the model's effectiveness. Furthermore, the hybrid architecture combining CNN and BiLSTM leverages the advantages of both techniques. This method holds promise for advancing research in therapeutic target identification and disease treatment.

Despite its strengths, the method presents certain limitations. The absence of an online web server hinders accessibility and user‐friendliness. Moreover, the omission of feature selection may impact the model's performance and efficiency. While not explicitly addressed, the potential for overfitting due to the complexity of deep learning models remains a concern. Additionally, the model's performance is contingent on the quality and representativeness of the training and testing datasets.

## 4 CONCLUSION

Globular proteins, characterised by their distinctive three‐dimensional structures, play crucial roles in diverse biological processes, ranging from enzymatic catalysis to immune responses. To achieve precise GP identification, this study introduces a novel approach employing a hybrid‐based deep learning model known as Deep‐GP.

The proposed model effectively addresses the challenge of GP prediction by integrating several innovative components. Firstly, the introduction of the CST‐PSSM feature descriptor effectively captures both global and local sequence information, enhancing feature representation compared to traditional PSSM‐based methods. Secondly, the hybrid architecture combining CNN and BiLSTM leverages the strengths of both deep learning techniques, resulting in improved predictive performance. The model's ability to outperform existing methods highlights its potential for advancing GP prediction research.

## AUTHOR CONTRIBUTION

**Sonia Zouari**: Data curation; Investigation. **Farman Ali**: Conceptualisation; Methodology. **Atef Masmoudi**: Writing ‐ original draft. **Sarah Abu Ghazalah**: Resources. **Wajdi Alghamdi**: Supervision. **Faris A. Kateb**: Software; Validation. **Nouf Ibrahim**: Writing ‐ review and editing.

## CONFLICT OF INTEREST STATEMENT

Authors have no competing interest.

## Data Availability

The datasets and code are available online freely at a public link: https://github.com/Farman335/Deep‐GB.
